# Mitochondrial Aminopeptidase Deletion Increases Chronological Lifespan and Oxidative Stress Resistance while Decreasing Respiratory Metabolism in *S. cerevisiae*

**DOI:** 10.1371/journal.pone.0077234

**Published:** 2013-10-08

**Authors:** Erine M. Stames, John F. O'Toole

**Affiliations:** Department of Medicine, Division of Nephrology MetroHealth Medical System and Case Western Reserve University School of Medicine, Cleveland, Ohio, United States of America; University of Texas Health Science Center at San Antonio, United States of America

## Abstract

Recessive mutations in *XPNPEP3*, encoding a mitochondrial x-prolyl aminopeptidase, have been identified in families with a rare hereditary tubulointerstitial kidney disease. The yeast ortholog of XPNPEP3, Icp55p, participates in the proteolytic processing and stabilization of mitochondrial proteins and its deletion accelerates the degradation of its protein targets. We used *icp55* deletion strains of *S. cerevisiae* to model loss of XPNPEP3 enzymatic function and study its phenotypic consequences on mitochondrial function. We found that Icp55p is not required for respiratory competence; however, compared to controls deletion strains had reduced mitochondrial oxygen consumption when grown in glucose containing media. The reduced mitochondrial respiration of *icp55* deletion strains in glucose media requires the mitochondrial peptide transporter, Mdl1p, and was corrected by Tor1p inhibition with rapamycin. Under similar growth conditions the abundance of the mitochondrial ATP synthase complex was decreased in the *icp55* deletion strain and was corrected by concurrent deletion of *tor1*. The *icp55* deletion strain demonstrated an increased chronological lifespan and decreased reactive oxygen species. These changes were additive to similar changes known to occur in *tor1* deletion strains suggesting independent mechanisms. Together, these results demonstrate that loss of Icp55p function reduces mitochondrial oxygen consumption and ATP synthase complex assembly in glucose media, while also promoting stress resistance, decreasing reactive oxygen species and increasing chronological lifespan through mechanisms that are distinct from decreased Tor1p activity.

## Introduction

We have recently identified mutations in the mitochondrial x-prolyl aminopeptidase, *XPNPEP3*, in families that have a rare autosomal recessive form of renal failure [1]. The renal histopathology includes interstitial fibrosis, tubular atrophy and tubular basement membrane disruption. *XPNPEP3* is conserved through evolution and its enzymatic function is inferred from studies performed with the *E. coli* ortholog, showing that it cleaves the amino-terminal residue of a peptide chain when proline appears in the second position [2]. Subsequent studies with the *S. cerevisiae* ortholog, intermediate cleaving peptidase 55 (Icp55p), confirmed that it has an aminopeptidase function, but the presence of proline in the second position of the peptide chain was not invariably required for cleavage of the amino-terminal residue [3], [4].

Both, XPNPEP3 in the human and Icp55p in yeast have been localized to the mitochondria [1], [3]–[6]. The large majority of mitochondrial proteins are encoded by nuclear genes, which is the case for *XPNPEP3*/*ICP55*. After translation in the cytosol many of these proteins are designated for mitochondrial import by an amino-terminal mitochondrial targeting signal. Following mitochondrial import, the mitochondrial targeting signal is removed by the mitochondrial processing peptidase (MPP) leaving a nascent amino-terminal residue [7]. Icp55p has recently been shown to contribute to the regulation of the half-life of its substrate mitochondrial proteins [4]. Deletion of *icp55* in *S. cerevisiae* increases the proteolytic rate of its substrate proteins through a protein degradation pathway characterized by the N-end rule [8], [9]. This proteolytic pathway utilizes the amino-terminal residue of a protein as a critical determinant of its degradation rate. Some amino acid residues facilitate the entry of proteins into the N-end protein degradation pathway when they appear at the amino-terminus and are known as destabilizing, others do not and are stabilizing. Peptide fragments resulting from the proteolysis of mitochondrial proteins may exit the mitochondria through a peptide transporter. Mdl1p is an ATP-binding cassette (ABC) transporter localized to the inner mitochondrial membrane of *S. cerevisiae* and has been shown to transport peptide fragments of degraded proteins from the mitochondrial matrix to the cytosol [10].

Mutations in many nuclear and mitochondrial genes have been implicated in the pathogenesis of mitochondrial diseases. A significant fraction lead to decrements in oxidative phosphorylation; however, most cases do not have a kidney phenotype and where renal involvement does occur, it is most often characterized by proximal tubular dysfunction [11]. While a defect in the respiratory chain did occur in one kindred in the original description of *XPNPEP3* mutations, this was not an invariant feature [1]. A proteomic study [4] has identified 38 targets of Icp55p that have diverse functional roles in the mitochondria. Deletion strains of *icp55* are viable, but do not grow on non-fermentable carbon substrates at 37°C. However, little else is known about the phenotypic impact of *icp55* deletion on mitochondrial function. The study of mitochondrial function in *S. cerevisiae* is facilitated by its ability to adapt its energy metabolism to the carbon substrate available in the culture media. *S. cerevisiae* utilize glucose in preference to most other fermentable carbon substrates (e.g., raffinose), and prefers fermentable over non-fermentable carbon substrates (*e*.*g*., glycerol) [12]. Fermentable carbon substrates can be metabolized in the absence of oxygen; whereas, non fermentable carbon substrates require the presence of oxygen and a functional mitochondrial respiratory chain. Therefore, *S. cerevisiae* cultured in glucose will metabolize glucose to ethanol through aerobic fermentation until glucose is depleted, then its metabolism is altered to consume ethanol through aerobic respiration in the mitochondria. Alternatively, when presented with a non-fermentable carbon substrate, such as glycerol, *S. cerevisiae* is forced to utilize aerobic respiration for cellular energy production. In addition, there is a precedent for using *S. cerevisiae* as a model organism for the study of human diseases with dysregulated protein homeostasis, most notably neurodegenerative disease [13]; therefore, we decided to use the yeast as a simple *in vivo* model to further characterize the functional role of the yeast ortholog of *XPNPEP3*.

## Materials and Methods

### Strains and reagents

*Icp55* deletion strains in the BY4741 (*MATa his3Δ1 leu2Δ0 met15Δ0 ura3Δ0*) and BY4742 (*MATα his3Δ1 leu2Δ0 lys2Δ0 ura3Δ0*) backgrounds were obtained (Open Biosystems) and deletions were confirmed with antibiotic selection and PCR based sequence confirmation. *tor1* and *mdl1* deletions in the BY4741 and *tor1* deletion BY4742 backgrounds with and without *Icp55* deletion were generated by replacing the ORF with a hygromycin resistance cassette, as previously described [14]. Plasmids for the generation of the targeted replacement cassettes were obtained from EUROSCARF [14]. All reagents were obtained from Fisher chemicals unless otherwise indicated and were of the highest purity available.

### Mitochondrial Oxygen consumption

YPG (1% yeast extract, 2% peptone, 2% glycerol) starter cultures were inoculated from independent single colonies and grown overnight at 30°C with shaking at 250 rpm. Batch cultures of the desired growth media were inoculated from these respiratory competent starter cultures with equivalent cell number as determined by OD_600_ and grown to late log phase at 30°C with shaking at 250 rpm. Cells were collected, wet weight of cell pellet was determined and resuspended in fresh growth media at 200 mg/ml and maintained at 30°C prior to sampling for determination of mitochondrial oxygen consumption. A baseline determination of oxygen concentration was made of 450 µl of fresh, air-saturated growth media equilibrated to 30°C in a thermostatically controlled Clark-type oxygen electrode (Qubit Systems Inc.) with constant stirring. After the addition of 50 µl (10 mg wet weight of yeast cells) the rate of oxygen consumption was recorded, 10 µl of 1 M sodium azide was then added to inhibit all mitochondrial oxygen consumption and mitochondrial-independent oxygen consumption rates were obtained. Finally, 50 µl of freshly prepared 1 M sodium dithionite was added to consume the remaining oxygen in the chamber and obtain the scale of measurement.

### Mitochondrial Isolation

For each replicate a YPG starter culture was inoculated from a single independent yeast colony. The starter culture was used to inoculate a 750 ml of YPD in a 2 L flask to an OD_600_ of 0.05 and incubated at 30°C with shaking at 250 rpm to late log phase (18 hours). Cells were harvested and mitochondria isolated using differential centrifugation yielding a crude mitochondrial fraction followed by density gradient centrifugation to obtain a pure mitochondrial fraction as described [15].

### Blue Native Gel Electrophoresis

The protein concentration of crude mitochondrial preparations were determined with DC protein assay kit (BioRad) and solubilized with digitonin (Sigma) using previously described detergent:protein ratios [16]. Mitochondrial proteins were separated on a NativePage 4–16% Bis-tris gel (Invitrogen) using native page buffer system according to manufacturer's instructions. Native Mark Unstained protein standards (Invitrogen) were used for protein size estimation. For Coomassie staining, gels were fixed by immersion in 100 ml fix solution (40% methanol, 10% acetic acid), heated on highest setting for 45 seconds in 1.55 kW microwave oven, followed by 15 minute room temperature incubation on orbital shaker. Fix solution was replaced with fresh solution and heating and incubation steps were repeated. Fix solution was replaced with stain solution (0.02% Coomassie R-250, 30% methanol, 10% acetic acid), heated in microwave 45 seconds and incubated with shaking at room temperature for 15 minutes. Stain solution was replaced with destain (8% acetic acid), heated for 45 seconds in microwave and incubated on orbital shaker. Destain was repeated twice. Images were captured as TIFF files and band densities were determined using ImageJ software available from the National Institutes of Health (http://rsb.info.nih.gov/ij/).

*In-gel Respiratory Complex Activity Assays*: Crude mitochondria isolated from parental strain BY4741 were prepared and separated with blue-native gel electrophoresis as described above. In-gel activity of respiratory complex V was determined on freshly run gels as previously described [17]. Briefly, gels were then incubated overnight in solution to detect complex V (35 mM Tris, 270 mM glycine, 14 mM MgSO_4_, 0.2% Pb(NO_3_)_2_, 8 mM ATP, pH = 7.8). After overnight incubation gels were briefly rinsed in deionized water and imaged as TIFF files and band densities were determined using ImageJ software available from the National Institutes of Health (http://rsb.info.nih.gov/ij/).

### Rapamycin resistance assay

Starter cultures, 10 mL YPG pH = 5, were inoculated from plates, and grown overnight at 30°C. These cultures were then used to inoculate 30 mL YPD cultures at a concentration of 10^6^ cells/mL. These cultures were grown to late phase. Each culture was adjusted to 10^7^ cells/mL, 10-fold serial dilutions were prepared and plated onto YPD plates containing 10 nM rapamycin (Sigma), from a 1 mg/ml stock solution, and control YPD plates prepared with an equal volume of vehicle (ethanol). Plates were incubated at 30°C for 48 hours.

### Flow Cytometry to determine reactive oxygen species

For each replicate starter cultures (synthetic minimal media with 2% dextrose) were inoculated from a single independent yeast colony and grown 24 hours. Batch cultures 25 ml of synthetic minimal media 2% dextrose were inoculated with 106 cells from each starter cultures and grown overnight 30°C, 250 rpm to equivalent cell densities as determined by OD_600_. Dihydroethidium (DHE) staining was performed as previously described [18] as follows, cells (2×10^6^) from each culture were prepared by resuspending in 100 µL of PBS with 50 µM DHE (Molecular Probes), and incubated at 30°C for 10 minutes. Cells were washed once in 1 mL PBS, and resuspended to a final volume of 400 µl PBS. An equivalent number of dead control cells, prepared by heating at 90°C for 10 minutes, were stained and analyzed in parallel with live cells. Flow cytometry was carried out on a Becton-Dickinson FACSCalibur model flow cytometer. DHE fluorescence was the direct output of the FL3 (red fluorescence-detecting) channel without compensation.

### Chronological Lifespan (CLS)

CLS experiments were performed using the yeast strain, BY4742 (*MATα his3Δ1 leu2Δ0 lys2Δ0 ura3Δ0*) to maximize the accuracy of the comparisons since it is relatively short-lived strain, as previously described [18]. Briefly, for each replicate, starter cultures (synthetic minimal media with 2% dextrose) were inoculated from a single independent yeast colony and grown overnight. Batch cultures 50 ml of synthetic minimal media 2% dextrose supplemented with histidine, leucine, lysine, and uracil were inoculated to obtain a starting cell density of cultures at 5×10^5^ cells/ml. Cultures were maintained at 30°C, while shaking at 250 rpm and cell density was measured daily by OD_600_ with a Beckman Coulter DU 800 spectrophotometer. Cell viability was determined daily on an aliquot sterilely removed from each culture by staining with 0.4% Trypan blue stain in a 1:1 volume of cells to stain solution then incubated at 30°C for 5 minutes to distinguish live dead status and cell counting with a hemocytometer. These results were corroborated by plating serial dilutions to ensure that results were qualitatively similar to the live/dead cell counts. The experiment was carried out over a period of 6 days.

### Assays for Resistance to Hydrogen Peroxide

Methods for assessing resistance to hydrogen peroxide were adapted from published methods [18]. For each replicate starter cultures of YPG (pH = 5) were inoculated from a single independent yeast colony and grown 24 hours. Starter cultures were used to inoculate 50 mL of synthetic minimal media with 2% dextrose at a density of 10^6^ cells/ml. After 24 hours 5 mL aliquots were removed from each cultures, and 100 µL were set aside for serial dilution and plating, while H_2_O_2_ was added to the remaining aliquot at 25 mM final concentration, and incubated for 2.5 hours. The serial dilutions of cells collected before and after H_2_O_2_ addition were plated on SD agar plates for viability comparison in addition to assessing cell viability with trypan blue staining as described above. These procedures were repeated after strains were cultured for 48 hours.

## Results

### Growth Fitness

**Figure 1:**
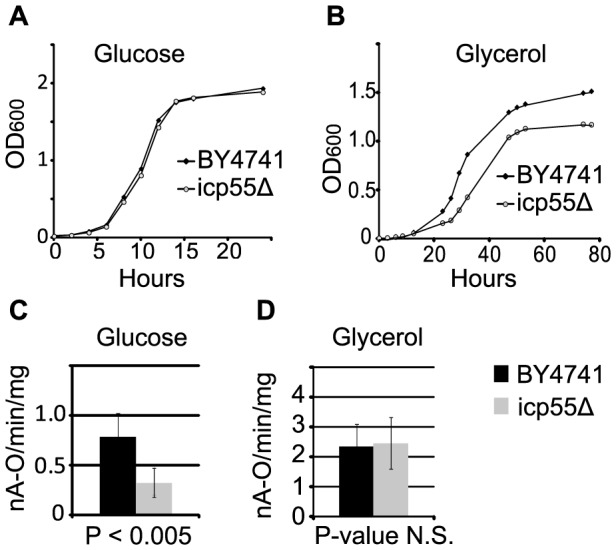
Growth rates and mitochondrial oxygen consumption of the *icp55Δ* strain with fermentable and non-fermentable carbon substrates. The growth rate of the *icp55Δ* strain was compared to BY4741 the parental strain from which it was derived in glucose (**A**), a fermentable carbon substrate, and glycerol (**B**), a non fermentable carbon substrate. Mitochondrial oxygen consumption was measured in the *icp55Δ* and BY4741 strains grown to late log phase in glucose (**C**) and glycerol (**D**) containing growth media. Growth curves were generated from five cultures inoculated from independent colonies. Assays of mitochondrial oxygen consumption were generated from cells collected from 6 independent cultures. Error bars represent standard deviation and p-values were calculated from two-tailed student t-test.

We began phenotyping the *Icp55* deletion (*icp55Δ*) strain by comparing its growth in media containing fermentable and non-fermentable carbon substrates with the haploid strain from which it was derived, BY4741. When grown in culture media containing fermentable carbon substrates *S. cerevisiae* utilize a combination of glycolysis and oxidative phosphorylation for energy production. When grown in non-fermentable carbon substrates *S. cerevisiae* is restricted to reliance on oxidative phosphorylation to supply cellular energy requirements. We found no difference in their growth curves when these strains were grown in glucose, a fermentable carbon substrate (**Figure 1A**). When grown in glycerol a non-fermentable carbon substrate we observed that *icp55Δ* strains reached stationary phase at a lower optical density (**Figure 1B**).

### Respiration

Since a central function of mitochondria is the generation of cellular energy we measured the rate of mitochondrial oxygen consumption in the *icp55Δ* strain compared to its parental haploid strain, BY4741. We found that the rates of mitochondrial oxygen consumption in both strains were comparable when energy production was limited to oxidative phosphorylation by supplying a single non-fermentable carbon substrate (*i*.*e*., glycerol) in the growth media (**Figure 1D**). Unexpectedly, the rate of mitochondrial oxygen consumption was reduced in the *icp55Δ* strain compared to the parental strain when glucose, a fermentable carbon substrate, was supplied as the sole carbon source in the growth media (**Figure 1C**).

### Blue Native Gel Electrophoresis and in-gel complex V activity assay

**Figure 2:**
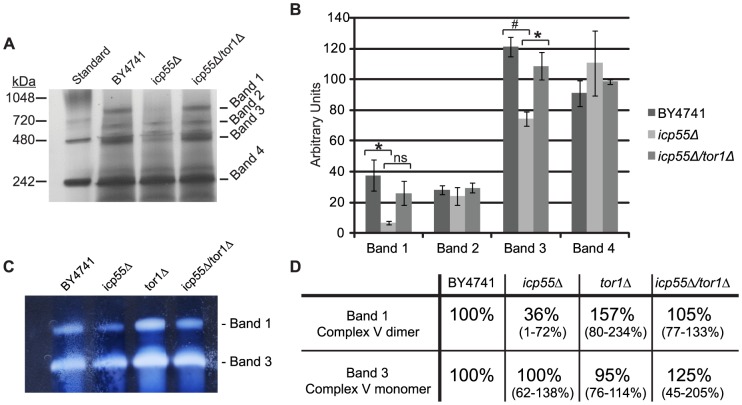
Decreased complex V abundance and activity in digitonin-solubilized mitochondria from the *icp55Δ* strain. Digitonin-solubilized mitochondria were separated on a 4–16% bis-tris gel using blue native gel electrophoresis (**A**). First lane contains size standards followed by equivalent amounts of mitochondrial protein. High molecular weight bands were quantitated (**B**) and average band density (n = 3) is reported in arbitrary units. Error bars represent standard error. P-values calculated from two-tailed student t-test brackets indicate pair-wise comparisons, asterisk p<0.05, number sign p<0.01, ns not significant. In-gel respiratory complex activity assays for complex V (CV), were performed on digitonin solubilized mitochondria (**C**) to confirm the identity of bands 1 (complex V dimer) and 3 (complex V monomer) shown in (**A**). These bands were quantitated to judge complex V activity and expressed as percent activity relative to the parental control and the 95% confidence interval is shown in parentheses below the normalized value.

We next examined isolated mitochondria from *icp55Δ* and parental strains grown in glucose containing media for differences in the assembled respiratory complexes and supercomplexes using blue-native gel electrophoresis. The solubilization of isolated mitochondria with the mild detergent, digitonin, allows the respiratory complexes to remain associated in supercomplexes which can then be separated under non-denaturing conditions with blue-native gel electrophoresis. The highest molecular weight supercomplexes isolated from the mitochondria of *S. cerevisiae* are complex V dimers (V_2_), complex III dimer with Complex IV dimer (III_2_/IV_2_), complex III dimer with a single complex IV (III_2_/IV), and complex V monomers [19]. Separation of digitonin solubilized mitochondria isolated from *icp55Δ* and BY4741 yeast demonstrate a significant reduction in two of these high molecular weight complexes (**Figure 2A and 2B**), that represent the ATP synthase dimer (band 1) and the ATP synthase monomer (band 3). Band densitometry shows that band 1 and band 3 are significantly reduced in the *icp55Δ* strain compared to the parental BY4741 strain when cultured in glucose containing media (**Figure 2B**). When grown in glycerol containing media the respiratory complexes were comparable in *icp55Δ* and BY4741 strains (**Supporting Information, Figure S1**), which is consistent with the similarity in mitochondrial oxygen consumption under these growth conditions (**Figure 1D**). Since loss of Tor1p function has been shown to increase mitochondrial oxygen consumption and respiration through the increased synthesis of components of the respiratory chain [20], [21], we speculated that deletion of *tor1* would be sufficient to correct the reduction in ATP synthase complexes noted in the *icp55Δ* strain. We found that combined deletion of *icp55* and *tor1* restored the ATP synthase monomers and dimers that were decreased in the strain with *icp55* deletion alone (**Figure 2A and 2B**). In-gel complex V activity assays (**Figure 2C**) were done to confirm that bands 1 and 3 (**Figure 2A and 2B**) are complex V dimers and monomers, respectively. Complex V in gel activity assays confirmed that the activity of complex V dimer was reduced in icp55*Δ* strains compared to the parental strain BY4741 (**Figure 2C**). The reduction of activity was expressed as a percentage of activity in each deletion strain compared to the parental strain with 95% confidence intervals (**Figure 2D**), The combined deletion strain, *icp55Δ/tor1Δ*, also restored complex V activity to levels comparable to the parental strain (**Figure 2C and 2D**).

### Tor1p inhibition increases mitochondrial oxygen consumption

**Figure 3:**
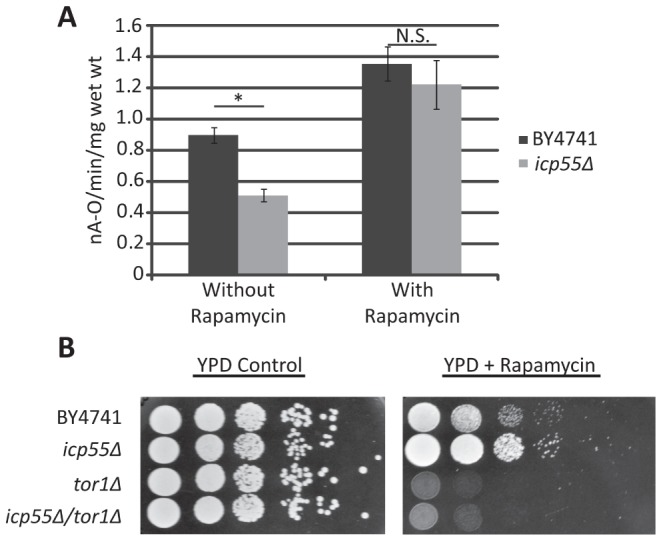
Inhibition of Tor1p corrects the reduction of mitochondrial oxygen consumption observed in the *icp55Δ* strain that has increased resistance to rapamycin. The parental BY4741 and *icp55Δ* strains were grown to late log phase in YPD (2% dextrose) (n = 25) an aliquot was removed from each culture and mitochondrial oxygen consumption rates were determined (**A**). Rapamycin was then added and cultures incubated an additional 4 hours followed by repeat determination of the mitochondrial oxygen consumption. Error bars represent standard error, p-values calculated with student's two tailed t-test. P-value before rapamycin represented by asterisk, 2.3×10^−7^, after rapamycin addition p-value was not significant (ns), 0.49. Cultures of BY4741, *icp55Δ*, *tor1Δ*, and *icp55Δ/tor1Δ* were grown to late log phase and serial dilutions were plated (**B**) on YPD with vehicle or YPD with 10 nM rapamycin and grown at 30°C for 48 hours.

Since deletion of *tor1* corrected the loss of ATP synthase complexes in the *icp55Δ* strain and is known to increase cellular oxygen consumption [20], we sought to determine if Tor1p inhibition would rescue the defect in oxygen consumption observed in the *icp55Δ* strain. Cells were grown to late log phase in glucose containing media, an aliquot was removed for determination of mitochondrial oxygen consumption and the remainder cultured four additional hours with rapamycin, an inhibitor of Tor1p, and prior to a final determination of mitochondrial oxygen consumption. As expected, the *icp55Δ* strain demonstrated a significantly reduced rate of mitochondrial oxygen consumption compared to the parental BY4741 strain prior to treatment with rapamycin (**Figure 3A**). However, after rapamycin was added to the same cultures and allowed to grow four more hours the rate of mitochondrial oxygen consumption in the *icp55Δ* strain increased and was comparable to that of the parental strain (**Figure 3A**). To determine if the *icp55Δ* strain had an increased Tor1p activity that may contribute to the repression of mitochondrial oxygen consumption we tested its resistance to rapamycin. We found that the *icp55Δ* strain demonstrated increased resistance to rapamycin in growth media compared to the parental strain (**Figure 3B**), consistent with the idea that *icp55* deletion increases Tor1p activity.

### Reduced mitochondrial oxygen consumption of the icp55Δ strain is corrected by mdl1 deletion

**Figure 4:**
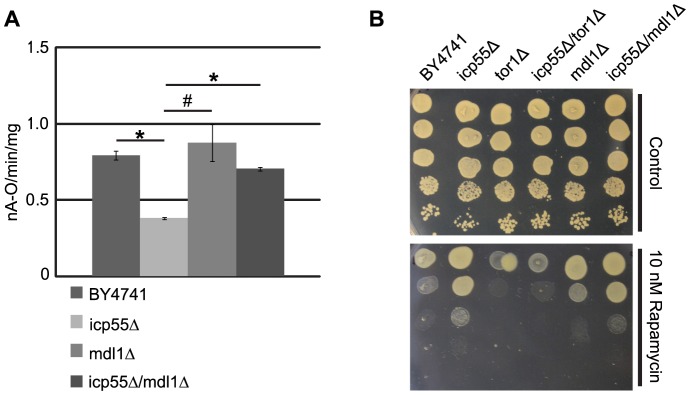
Deletion of mitochondrial peptide transporter corrects decreased mitochondrial oxygen consumption of the *icp55Δ* strain. Mitochondrial oxygen consumption was assayed in strains with deletion of *icp55*, *mdl1* and both together compared to the parental BY4741 strain (**A**). The rate of oxygen consumption was decreased in the *icp55Δ* strain compared to BY4741, *mdl1Δ* and *icp55Δ/mdl1Δ* with p-values of 0.054 (#) and <0.01 (*), all other comparisons were not significant. The bar graph depicts the average of three experiments determining the rate of oxygen consumption for each strain. Error bars depict standard error. P-values were calculated using a two-tailed student's t-test. Cultures of BY4741, *icp55Δ*, *tor1Δ*, *icp55Δ/tor1Δ*, *mdl1Δ*,and *icp55Δ/mdl1Δ* were grown to late log phase and serial dilutions were plated (**B**) on YPD with vehicle or YPD with 10 nM rapamycin and grown at 30°C for 48 hours.

Since the reduction in mitochondrial oxygen consumption only occurred in glucose containing media and was correctable with Tor1p inhibition we reasoned that loss of Icp55p function was sensed by the cell and enhanced the glucose repression of mitochondrial oxygen consumption. We hypothesized that loss of Icp55p function would increase mitochondrial protein degradation, and the efflux of degraded peptide fragments from the mitochondria to the cytosol may be the signal mediating this effects. The mitochondria of *S. cerevisiae* contain an ATP-binding cassette (ABC) protein that is known to transport peptides from the mitochondria, Mdl1p [10]. When we deleted *mdl1* alone there was no effect on the rate of mitochondrial oxygen consumption; however, when combined with *icp55* deletion, the decrement in mitochondrial oxygen consumption of the *icp55Δ* strain was corrected (**Figure 4A**). We suspected that peptides exported from the mitochondria through Mdl1p may stimulate Tor1p activity in the *icp55Δ* strain; thereby, mediating the increased rapamycin resistance observed in this strain (**Figure 3B**). We expected that the combined deletion of mdl1 with icp55 would reduce the rapamycin resistance of the *icp55Δ* strain to the level observed in the parental strain. However, we found that rapamycin resistance in the combined deletion of *mdl1* with *icp55* was comparable to the *icp55Δ* strain (**Figure 4B**), suggesting that the increased Tor1p activity in the *icp55Δ* strain is not mediated by efflux of peptide fragments through Mdl1p.

### Chronological lifespan extension

**Figure 5:**
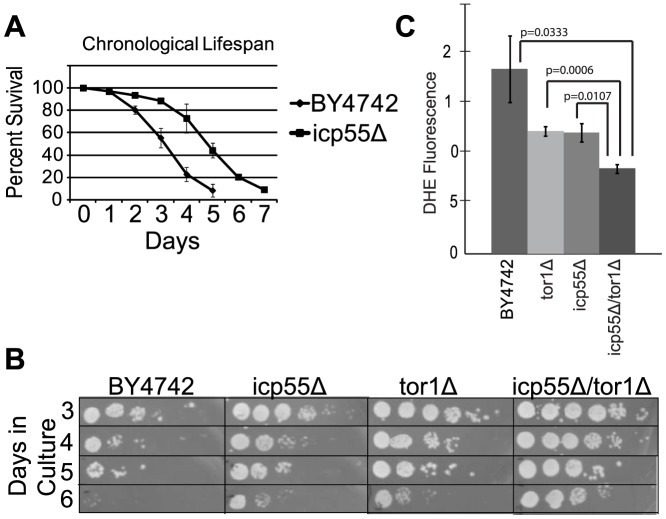
Increased chronological lifespan and decreased reactive oxygen species in the *icp55Δ* strain. The chronological lifespan of the *icp55Δ* strain was compared to the short-lived parental strain, BY4742 (**A**) through measurement of percent survival by trypan blue staining (n = 3) each strain. The chronological lifespan of the short-lived parental strain, BY4742 was compared to the strains *icp55Δ*, *tor1Δ* or *icp55Δ*/*tor1Δ* combined by plating serial dilutions of cells maintained in culture (n = 3 for each strain) on sequential days (**B**), representative pictures. Dihydroethidium staining of live cells grown to early stationary phase in glucose media was measured with flow cytometry (n = 3 for each strain) to evaluate superoxide production (**C**). Error bars (**A** and **C**) represent standard deviation; p-values (**C**) were calculated using student's two-tailed t-test.

The repression of TOR activity is well known to increase lifespan in many species from mice to yeast [22]. Chronological lifespan (CLS) is a measure of the length of time that yeast cells remain viable in culture after reaching stationary phase and are no longer dividing. Maneuvers that impair mitochondrial respiratory metabolism also inhibit the CLS extension observed in *Tor1* deletion strains [20], [21] and its downstream target, Sch9p, a functional ortholog of the S6 kinase 1 in mammals [23]. We anticipated that the *icp55Δ* strain would have a reduced CLS and therefore, measured the CLS of *icp55* deletion in comparison to *tor1* deletion and the short-lived parental yeast strain, BY4742. However, we found that the *icp55Δ* strain had an increased CLS compared to the parental strain (**Figure 5A**). The CLS extension observed in *icp55Δ* strains was comparable to that of *tor1Δ* strains, which is known to extend CLS (**Figure 5B**). In addition, we found that the combined deletion of *tor1* and *icp55* resulted in an additive increase in CLS beyond that observed when either gene was deleted alone (**Figure 5B**).

### Reactive oxygen species production and hydrogen peroxide resistance

In stationary phase *tor1* deletion has been reported to increase resistance to hydrogen peroxide and decrease reactive oxygen species production [20]. We speculated that loss of Icp55p function may activate stress response pathways, increasing resistance to reactive oxygen species (ROS). We found that ROS measured by flow cytometry in the *icp55Δ* strain was comparable to the *tor1* deletion strain (**Figure 5C**) and the combined deletion of *icp55* and *tor1* led to an additive reduction in ROS. Similarly, the deletion of either *icp55* or *tor1* increased resistance to hydrogen peroxide and their combined deletion (data not shown) led to an increase in resistance beyond that observed for either gene alone.

## Discussion

*ICP55* is the yeast ortholog of *XPNPEP3* a gene mutated in a rare hereditary kidney disease resembling nephronophthisis with renal histopathology including tubular atrophy, tubular basement disruption and interstitial fibrosis. The gene product of *ICP55/XPNPEP3* is an aminopeptidase [2]–[4] that is localized to the mitochondria [1], [3]–[6]. Icp55p has been implicated in the post-translational processing of proteins imported into the mitochondria contributing to their protein half-life determination via the N-end rule protein degradation pathway [4]. In this report, we have utilized *S. cerevisiae* as a simple model organism to characterize the phenotype of the *icp55* deletion (*icp55Δ*) strain and gain insight into the pathogenetic mechanisms that contribute to the progressive kidney disease observed in the setting of *XPNPEP3* mutations.

We first examined the effect of *icp55* deletion on mitochondrial respiration by taking advantage of the fact that *S. cerevisiae* will adapt its energy metabolism to the carbon substrate available in the culture media as discussed in the introduction. We found that the *icp55Δ* strain is viable, and Icp55p function is not required for the proper assembly and function of the respiratory chain, demonstrated by the similar rates of mitochondrial oxygen consumption in *icp55Δ* strains and parental strains when cultured in glycerol, a non-fermentable carbon substrate that limits cellular energy production to mitochondrial respiration (**Figure 1D**). However, we did observe reduced mitochondrial respiration in *icp55Δ* compared to the parental strain when cultured in glucose media (**Figure 1C**). This reduction in mitochondrial oxygen consumption was accompanied by a decrease in the abundance and activity of ATP synthase (complex V of the respiratory chain) in isolated mitochondria of the *icp55Δ* strain compared to the parental strain (**Figure 2A and B**). The delta (Atp16p) and gamma (Atp3p) subunits of the F1 component of ATP synthase (complex V) and Atp11p a chaperone for the assembly of the F1 component have previously been identified as substrates of Icp55p [4]. As substrates, their protein half-life in the mitochondria should be shorter and may contribute to the decreased oxygen consumption and complex V abundance observed in *icp55Δ* strains grown in glucose containing media. Many genetic constituents of ATP synthase are subject to glucose repression [24] and we suspect that *icp55* deletion combined with glucose repression may be responsible for the decreased abundance of ATP synthase in the *icp55Δ* strain.

Since the deletion of *tor1* is known to increase the expression of respiratory chain proteins and increase mitochondrial respiration [20], [21], we speculated that *tor1* deletion may restore complex V activity and mitochondrial respiration in the *icp55Δ* strain. The combined deletion of *icp55* and *tor1* corrected the decreased abundance and activity of complex V in the *icp55Δ* strain (**Figure 2**). We also showed that rapamycin inhibition of Tor1p corrected the decrease in mitochondrial oxygen consumption in the *icp55Δ* strain and the *icp55Δ* strain demonstrated increased resistance to rapamycin (**Figure 3**). These results are consistent with the hypothesis that loss of Icp55p function stimulates Tor1p activity in *S. cerevisiae*.

As others have shown that *icp55* deletion increased mitochondrial protein degradation [3], [4], we speculated that peptide fragments transported from the mitochondria to the cytosol may serve as a signal to stimulate Tor1p activity in the setting of *icp55* deletion. Supporting this hypothesis, we demonstrated that deletion of *mdl1*, a mitochondrial peptide transporter, with *icp55* corrected the decrease of mitochondrial oxygen consumption observed in the *icp55Δ* strain (**Figure 4A**). However, the persistent rapamycin resistance of the *icp55Δ* strain in spite of concurrent *mdl1* deletion (**Figure 4B**) suggests that Tor1p is not activated by peptide efflux through Mdl1p in the *icp55Δ* strain, but through an alternative mechanism.

The reduction of mitochondrial respiration in glucose containing media is a complex phenotype. Our results show that it can be corrected by the deletion of either *tor1* or *mdl1* in combination with *icp55*, suggesting that several mechanisms are capable of attenuating this phenotype in the *icp55Δ* strain and emphasizing the complexity of this phenotype. The mechanism underlying this phenotype in the *icp55Δ* strain remains uncertain, and possibilities include increased glucose repression of respiratory metabolism, improper assembly or accelerated degradation of the respiratory chain. In addition, we do not believe that this mitochondrial respiratory defect is likely to be the central mechanism mediating progression of XPNPEP3-related kidney damage for several reasons. First, a defect in mitochondrial respiratory function was not an invariant feature in the setting of *XPNPEP3* mutation [1]. Second, there are many examples of genetic mitochondriopathies impairing respiratory chain function that do not have a renal phenotype [11]. Third, whereas Tor1p activity decreases mitochondrial respiration in *S. cerevisiae* [20], [21] by suppressing the synthesis of respiratory chain components, mTOR signaling increases mitochondrial respiration in mammalian cells [25]–[27]. Therefore, we suspect that a more likely contributor to disease pathogenesis may be related to mTOR activation. The upregulation of Tor1p/mTORC1 activity has been associated with several kidney pathologies including acute kidney injury, diabetic nephropathy and cystic kidney diseases [28].

Since these results suggest an increased Tor1p activity in the *icp55Δ* strain and inhibition of Tor1p activity or deletion of *tor1* has been shown to increase the chronological lifespan (CLS) of yeast, we anticipated that the *icp55Δ* strain would have a decreased CLS. We were surprised to find that compared to the parental wild-type strain the *icp55Δ* strain had an increase in CLS, comparable to that observed for the *tor1Δ* strain. However, when *icp55* and *tor1* were deleted in combination the increase in CLS was additive, suggesting that the mechanism for CLS extension due to each gene is independent. Similarly, the *tor1Δ* strain had decreased reactive oxygen species (ROS) and increased resistance to hydrogen peroxide, as previously reported [20], which was comparable to the *icp55Δ* strain and the deletion of both *icp55* and *tor1* resulted in an additive decrease of ROS and increase of H_2_O_2_ resistance, again suggesting independent, parallel mechanisms of stress resistance.

Taken together these observations suggest that mitochondrial signaling to the cell body in the *icp55Δ* strain is increasing Tor1p activity and activating stress resistance pathways that are independent of TOR. The activation of Tor1p/mTORC1 by amino acids is well recognized from yeast to mammals [29], [30], and small GTPases have been identified as important mediators of this activation. However, the rapamycin resistance observed in the *icp55Δ* strain persisted with the combined deletion of *icp55* with *mdl1*, a transporter of peptide fragments from the mitochondrial matrix [10], suggesting that the mechanism of Tor1p activation in the *icp55Δ* strain is not through the efflux of peptides from the mitochondria and remains an attractive area for future studies.

In addition to the activation of Tor1p suggested by rapamycin resistance, the extension of chronological lifespan and reduction of ROS suggest that loss of icp55p function initiates a mitochondrial signal mediating these phenotypes. This type of mitochondrial signaling may be analogous to the mitochondrial unfolded protein response that has been best characterized in the nematode, *C. elegans*, reviewed in [31], in which mitochondrial proteases and peptide transporters have been implicated. Mitochondrial stress, such as the unfolded protein response, may also modify the import of proteins into the mitochondria; where peptides exported from the mitochondria through peptide transporters decrease the import of nuclear encoded mitochondrial proteins [32]. When the import of mitochondrial proteins is reduced in the setting of mitochondrial stress such as the unfolded protein response, a key transcription factor is relocalized from the mitochondria to the nucleus mediating the mitochondrial stress response [32]. Recent data has also linked the uncoordinated expression of mitochondrial proteins encoded in the nucleus and mitochondria with a mechanism of lifespan extension that is conserved across species [33].

It may seem counterintuitive that the deletion of a gene implicated in renal failure would lead to apparent benefits such as increased stress resistance or increased chronological lifespan. However, it should be emphasized that these apparent benefits are occurring under specified laboratory conditions and in an uncontrolled environment loss of Icp55p function may result in decreased competitive fitness. Similarly, it might be imagined that in higher organisms compensatory adaptations to environmental or genetic stressors may lead to short-term benefits at the cost of damage that occurs over longer time intervals. These experiments may form the basis for further investigations in higher organisms and provide insight into relevant mechanisms in the pathogenesis of tubulointerstitial kidney diseases.

## Supporting Information

Figure S1 **Blue Native Gel Separation of mitochondrial respiratory supercomplexes from *icp55Δ* and BY4741 yeast strains cultured in glycerol containing media.** The parental yeast strain BY4741 and the *icp55Δ* strain were cultured in glycerol containing media restricting energy metabolism to oxidative phosphorylation. Mitochondria were isolated with differential centrifugation, digitonin-solubilized and separated on a 4–16% bis-tris gel using blue native gel electrophoresis. Two replicates of each strain were examined and demonstrated comparable abundance of the respiratory complexes, labeled at the right-hand side of the second gel (arrowheads designate bands containing complex V components and asterisks designate complex III dimers with complex IV monomers or dimers) (**A**). The migration pattern of respiratory complexes from digitonin solubilized mitochondria of the parental strain (BY4741) were characterized by separation on a 4–16% bis-tris gel with molecular weight standards (Stand) and coomassie staining (**B**). In-gel activity assays of complex IV (CIV) labeled the complex III dimers with complex IV monomers or dimers with a pale brown precipitate, indicated by asterisks (**B**). In-gel activity assays of complex V on a light background (CV light) and dark background (CV dark) labeled complex V dimers and monomers with a white precipitate, indicated by arrowheads (**B**).
(EPS)

## References

[1] O'Toole JF, Liu Y, Davis EE, Westlake CJ, Attanasio M, et al (2010) Individuals with mutations in XPNPEP3, which encodes a mitochondrial protein, develop a nephronophthisis-like nephropathy. J Clin Invest 120: 791–802. 20179356 10.1172/JCI40076 PMC2827951

[2] Yoshimoto T, Murayama N, Honda T, Tone H, Tsuru D (1988) Cloning and expression of aminopeptidase P gene from Escherichia coli HB101 and characterization of expressed enzyme. J Biochem 104: 93–97. 2851590 10.1093/oxfordjournals.jbchem.a122430

[3] Naamati A, Regev-Rudzki N, Galperin S, Lill R (2009) Dual targeting of Nfs1 and discovery of its novel processing enzyme, Icp55. J Biol Chem 284: 30200–30208. 19720832 10.1074/jbc.M109.034694 PMC2781575

[4] Vogtle FN, Wortelkamp S, Zahedi RP, Becker D, Leidhold C, et al (2009) Global analysis of the mitochondrial N-proteome identifies a processing peptidase critical for protein stability. Cell 139: 428–439. 19837041 10.1016/j.cell.2009.07.045

[5] Huh WK, Falvo JV, Gerke LC, Carroll AS, Howson RW, et al (2003) Global analysis of protein localization in budding yeast. Nature 425: 686–691. 14562095 10.1038/nature02026

[6] Reinders J, Zahedi RP, Pfanner N, Meisinger C, Sickmann A (2006) Toward the complete yeast mitochondrial proteome: multidimensional separation techniques for mitochondrial proteomics. J Proteome Res 5: 1543–1554. 16823961 10.1021/pr050477f

[7] Mossmann D, Meisinger C, Vogtle FN (2012) Processing of mitochondrial presequences. Biochim Biophys Acta 1819: 1098–1106. 22172993 10.1016/j.bbagrm.2011.11.007

[8] Bachmair A, Finley D, Varshavsky A (1986) In vivo half-life of a protein is a function of its amino-terminal residue. Science 234: 179–186. 3018930 10.1126/science.3018930

[9] Varshavsky A (2011) The N-end rule pathway and regulation by proteolysis. Protein Sci 10. 10.1002/pro.666 PMC3189519 21633985

[10] Young L, Leonhard K, Tatsuta T, Trowsdale J, Langer T (2001) Role of the ABC transporter Mdl1 in peptide export from mitochondria. Science 291: 2135–2138. 11251115 10.1126/science.1056957

[11] Niaudet P (1998) Mitochondrial disorders and the kidney. Arch Dis Child 78: 387–390. 9623410 10.1136/adc.78.4.387 PMC1717549

[12] Broach JR (2012) Nutritional control of growth and development in yeast. Genetics 192: 73–105. 22964838 10.1534/genetics.111.135731 PMC3430547

[13] Tenreiro S, Outeiro TF (2010) Simple is good: yeast models of neurodegeneration. FEMS Yeast Res 10: 970–979. 20579105 10.1111/j.1567-1364.2010.00649.x

[14] Janke C, Magiera MM, Rathfelder N, Taxis C, Reber S, et al (2004) A versatile toolbox for PCR-based tagging of yeast genes: new fluorescent proteins, more markers and promoter substitution cassettes. Yeast 21: 947–962. 15334558 10.1002/yea.1142

[15] Meisinger C, Pfanner N, Truscott KN (2006) Isolation of yeast mitochondria. Methods Mol Biol 313: 33–9 – 16118422 10.1385/1-59259-958-3:033

[16] Wittig I, Braun HP, Schagger H (2006) Blue native PAGE. Nat Protoc 1: 418–428. 17406264 10.1038/nprot.2006.62

[17] Nijtmans LG, Henderson NS, Holt IJ (2002) Blue Native electrophoresis to study mitochondrial and other protein complexes. Methods 26: 327–334. 12054923 10.1016/S1046-2023(02)00038-5

[18] Bonawitz ND, Rodeheffer MS, Shadel GS (2006) Defective mitochondrial gene expression results in reactive oxygen species-mediated inhibition of respiration and reduction of yeast life span. Mol Cell Biol 26: 4818–4829. 16782871 10.1128/MCB.02360-05 PMC1489155

[19] Schagger H, Pfeiffer K (2000) Supercomplexes in the respiratory chains of yeast and mammalian mitochondria. EMBO J 19: 1777–1783. 10775262 10.1093/emboj/19.8.1777 PMC302020

[20] Bonawitz ND, Chatenay-Lapointe M, Pan Y, Shadel GS (2007) Reduced TOR signaling extends chronological life span via increased respiration and upregulation of mitochondrial gene expression. Cell Metab 5: 265–277. 17403371 10.1016/j.cmet.2007.02.009 PMC3460550

[21] Pan Y, Shadel GS (2009) Extension of chronological life span by reduced TOR signaling requires down-regulation of Sch9p and involves increased mitochondrial OXPHOS complex density. Aging (Albany NY) 1: 131–145. 20157595 10.18632/aging.100016 PMC2815770

[22] Pan Y, Nishida Y, Wang M, Verdin E (2012) Metabolic regulation, mitochondria and the life-prolonging effect of rapamycin: a mini-review. Gerontology 58: 524–530. 22947849 10.1159/000342204

[23] Urban J, Soulard A, Huber A, Lippman S, Mukhopadhyay D, et al (2007) Sch9 is a major target of TORC1 in Saccharomyces cerevisiae. Mol Cell 26: 663–674. 17560372 10.1016/j.molcel.2007.04.020

[24] Westergaard SL, Oliveira AP, Bro C, Olsson L, Nielsen J (2007) A systems biology approach to study glucose repression in the yeast Saccharomyces cerevisiae. Biotechnol Bioeng 96: 134–145. 16878332 10.1002/bit.21135

[25] Schieke SM, Phillips D, McCoy JP Jr, Aponte AM, Shen RF, et al (2006) The mammalian target of rapamycin (mTOR) pathway regulates mitochondrial oxygen consumption and oxidative capacity. J Biol Chem 281: 27643–27652. 16847060 10.1074/jbc.M603536200

[26] Ramanathan A, Schreiber SL (2009) Direct control of mitochondrial function by mTOR. Proc Natl Acad Sci U S A 106: 22229–22232. 20080789 10.1073/pnas.0912074106 PMC2796909

[27] Cunningham JT, Rodgers JT, Arlow DH, Vazquez F, Mootha VK, et al (2007) mTOR controls mitochondrial oxidative function through a YY1-PGC-1alpha transcriptional complex. Nature 450: 736–740. 18046414 10.1038/nature06322

[28] Lieberthal W, Levine JS (2012) Mammalian target of rapamycin and the kidney. II. Pathophysiology and therapeutic implications. Am J Physiol Renal Physiol 303: F180–F191. 22496407 10.1152/ajprenal.00015.2012

[29] Kim J, Guan KL (2011) Amino acid signaling in TOR activation. Annu Rev Biochem 80: 1001–32 – 21548787 10.1146/annurev-biochem-062209-094414

[30] Lieberthal W, Levine JS (2012) Mammalian target of rapamycin and the kidney. I. The signaling pathway. Am J Physiol Renal Physiol 303: F1–10. 22419691 10.1152/ajprenal.00014.2012

[31] Haynes CM, Ron D (2010) The mitochondrial UPR - protecting organelle protein homeostasis. J Cell Sci 123: 3849–3855. 21048161 10.1242/jcs.075119

[32] Nargund AM, Pellegrino MW, Fiorese CJ, Baker BM, Haynes CM (2012) Mitochondrial import efficiency of ATFS-1 regulates mitochondrial UPR activation. Science 337: 587–590. 22700657 10.1126/science.1223560 PMC3518298

[33] Houtkooper RH, Mouchiroud L, Ryu D, Moullan N, Katsyuba E, et al (2013) Mitonuclear protein imbalance as a conserved longevity mechanism. Nature 497: 451–457. 23698443 10.1038/nature12188 PMC3663447

